# Linking Generalized Ligamentous Laxity to Musculoskeletal Injury: A Study in the Indian Population

**DOI:** 10.7759/cureus.52180

**Published:** 2024-01-12

**Authors:** Tirthankar Dasgupta, Tushar Gogia, Lalit Mohan Gupta, Kumud Kishlaya, Rahul Garg, Nitin Sahu

**Affiliations:** 1 Orthopaedics, Base Hospital Delhi Cantt, New Delhi, IND; 2 Orthopaedics, Command Hospital Western Command, Chandigarh, IND

**Keywords:** beighton score, generalized ligamentous laxity, indian population, hypermobility, musculoskeletal injury

## Abstract

Introduction

Generalized ligamentous laxity (GLL) is defined as an increased range of motion across multiple joints in an individual beyond the mean range of motion in the general population, with a reported prevalence between 5% and 15%. It becomes less common with age and is more common in females and in the lower limbs. Musculoskeletal injury (MSI) is damage to musculoskeletal systems, usually due to strenuous activity. There is conflicting literature regarding whether the risk of MSI during strenuous activity is higher in individuals with GLL and a dearth of evidence from the Indian subcontinent regarding GLL. This study determines if GLL predisposes to musculoskeletal injuries among patients.

Materials and methods

One hundred eighty patients each were selected as cases and controls after obtaining informed consent, a Beighton score assessment, and a questionnaire regarding injury-filled in all participants with GLL.

Result

Thirty-three participants (18.33%) in the case group and 16 participants (8.89%) in the control group were found to have GLL. An odds ratio of 2.30 (using a 2x2 RC table) was calculated between participants with GLL among the cases and controls with a Beighton score of 4/9, and a significantly higher mean Beighton score (p=0.018) was found among participants presenting with MSI (cases) than participants without MSI (controls).

Discussion

The study found that there was a significant prevalence of GLL in the adult population, especially in females compared to males. The younger age group was also comparatively much more involved. It also proved that GLL was more common in patients with MSI and that hyper-mobile people had a twofold chance of injury compared to the general population. The joints of the lower limb were more frequently involved, probably the weight-bearing joints, the most common being the ankle and knee. People with GLL also had higher chances of repeating injuries.

Conclusion

This study has implications for the prevention of injuries in people with GLL. Screening such individuals to identify those with GLL using the Beighton score could be beneficial. Though orthopedic surgeons primarily manage people with MSI, they rarely identify individuals with GLL, and making a diagnosis regarding the same definitely helps these individuals live pain-free lives.

## Introduction

Ligaments are one of the most important passive restraints on synovial joints. Laxity or looseness of ligaments invariably leads to an increase in the range of movement of the joint, or hypermobility.

Generalized ligamentous laxity (GLL) is defined as the increased range of motion or hypermobility in more than one joint as compared to the normal by Beighton and Graham [1]; colloquially, those affected are known as “loose jointed” or “double jointed.” GLL is an entity that results in joint hypermobility. It is seen in patients in whom a genetic disorder affects the connective tissue. It may be syndromic as well, e.g., trisomy 21, Marfan and Ehler-Danlos syndromes, etc. [2]. However, in most of the patients, the hypermobility is isolated. In these individuals, where there is no readily identified genetic aberrancy, it has been labeled as GLL [3].

The first clinical description of articular hypermobility is attributed to Hippocrates, who, in the fourth century B.C., described the Scythians, a race of Iranian horse-riding nomads, as having humidity, flabbiness, and atony such that they were unable to use their weapons [1]. Thereafter, the study of joint hypermobility took to the background, and patients were ignored. Until the early nineteenth century, when general physicians found many syndromes affecting connective tissue matrix proteins, which included GLL of various severity as an important feature that includes the Ehlers-Danlos syndrome, Marfan syndrome, and osteogenesis imperfecta, it became more and more of an interest [1]. It was concluded that it is associated with abnormal collagen and elastin. In GLL, the collagen fibrils were found to be thin and twisted [4].

Musculoskeletal injury (MSI) is the damage that occurs to the muscular or skeletal systems as a result of repetitive and strenuous activity [5].

The Beighton score is the standard measure of joint laxity. The score is on an ordinal scale from 0 to 9, with a higher score representing greater GLL [4]. A score of ≥4 indicates increased joint laxity. The Beighton test is highly reliable and reproducible, and hence it has been chosen to identify generalized joint laxity in this study.

There had been undeniable evidence from a review of the literature that Asians and Indians, by extension, showed a relatively higher prevalence of hypermobility compared to the Western population. In 1970, Schweitzer, in a comparative inter-racial study at Cape Town, reported that on comparison of Indians, Africans, and Europeans, Indians demonstrated maximum laxity and Europeans the least [6]. Wordsworth et al. compared English Caucasian subjects with Asian Indians in a group of patients suffering from a variety of inherited disorders and found Asian Indians to be significantly more hypermobile than English Caucasians [7]. In a recent study in North America by Russek et al., among 267 healthy college students, a significantly higher prevalence of GLL was found in Asians (25.3%) compared to North American Caucasians [8].

Despite the probability of increased GLL affliction in the Indian population, only a few studies into GLL were found during the literature search. Most of these were incidence studies conducted in the pediatric age group. A study conducted by Mullick et al. in 2010 at Army Hospital (Referral and Research), Delhi, found GLL in 5.8% (145/2468 patients reporting to OPD, using the Beighton score) with a mean Beighton score of 6/9 (range: 4/9-9/9) [9]. In 2001, Kumar et al. reported an incidence of 20% among 2050 patients reporting to the rheumatology orthopedic outpatient department (OPD) at All India Institute of Medical Sciences, New Delhi, with the knee being the most commonly involved joint, followed by the hand, elbow, ankle, and shoulder [10].

The objective of this study was to determine if GLL may be a predisposing factor in patients visiting an OPD for musculoskeletal injuries by comparing the prevalence of GLL in patients visiting for musculoskeletal injuries (cases) with age and gender-matched controls visiting for non-orthopedic complaints (controls).

## Materials and methods

This case-control study was performed at the Armed Forces Medical College, Pune, India, over a period of two years (July 2019-June 2021). The Institutional Ethics Committee of the Armed Forces Medical College approved the study (approval number: IEC/AFMC/09/2019).

The sample size of 180 participants (rounded off from 176) each in cases and controls was calculated using the two-tailed proportion formula for case-control studies. Consenting participants ages 15-60 were chosen. Pregnancy or a known history of primary connective tissue or syndromic disorder such as trisomy 21, Marfans and Ehler-Dahlos syndromes, etc. were excluded. To eliminate age and sex as confounding factors and increase the sensitivity of the statistical analysis, stratification was used based on sex and age groups.

Cases and controls

Patients with a definite history of MSI and presenting to the orthopedics OPD with complaints including pain, swelling, restriction of range of movement, instability, and inability to return to the previous level of physical activity were considered the pool of cases for the study. Any acute injury resulting in a fracture/dislocation (in need of urgent medical attention) was excluded.

Patients without any pain in or around joints and no history of MSI visiting the medical or surgical OPD were considered as the pool of controls for the study.

Collection of data

After informed consent, patients were asked to fill out a questionnaire. Two blinded clinician observers assessed the Beighton scores of all participants. A cut-off score of 4 or greater was used to determine the presence of GLL. Only participants with the presence of GLL (in both cases and the control group) were asked about earlier musculoskeletal injuries, joints involved, and associated complaints in the last two years. In addition, participants in the case group were asked about the joints in which the subjects suffered injuries. If the participants suffered an injury to the same joint again, it was termed a recurrent injury. The odds ratio was calculated using a 2x2 RC table.

## Results

A total of 465 subjects were approached for the study, which included 212 for the case group and 253 for the control group. One hundred forty-six patients were not included in the study as they did not meet the inclusion criteria and the requisite number for their age group was already achieved. Out of the 360 subjects included in the study, 180 were male and 180 were female. Stratification into six sub-groups in cases and six sub-groups in the control group with 30 participants each helped to ensure no significant differences were found in number, sex, and age between cases and controls. The distribution of demographic information for the study groups is provided in Table 1.

**Table 1:** Patient demographics

Description	Cases	Controls	P-value
Total number of participants	180	180	1.0
Total males	90	90	1.0
Total females	90	90	1.0
Mean age in years (+/-SD)	37.64 (13.84)	36.93 (13.75)	0.626
Mean age 15-30 years males	20.90 (4.29)	22.23 (4.82)	0.264
Mean age 15-30 years females	22.40 (4.88)	20.40 (4.08)	0.091
Mean age 31-45 years males	37.77 (4.48)	37.63 (3.79)	0.896
Mean age 31-45 years females	37.93 (5.42)	35.27 (5.19)	0.059
Mean age 46-60 years males	53.70 (5.15)	53.03 (4.33)	0.588
Mean age 46-60 years females	53.13 (4.28)	53.00 (4.66)	0.937

The average BMI of the participating population was 24.71 kg/m2 (SD±4.20), with no significant differences between cases and controls. However, there was an increasing trend in average BMI with age. No significant differences in activity level were found between the group and sub-groups (p>0.05). A decrease in activity levels was observed with increasing age.

**Figure 1:**
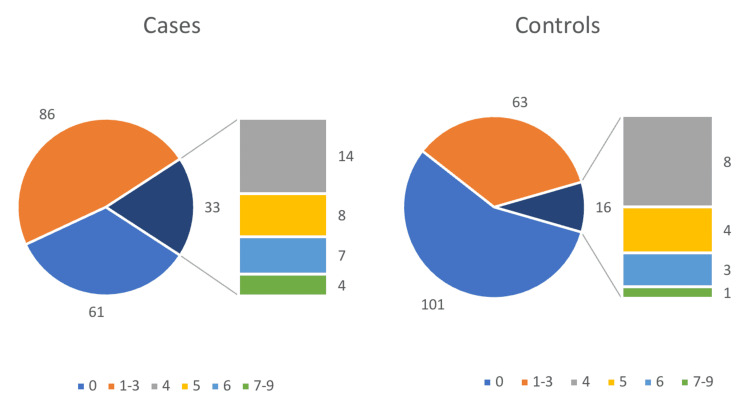
Presence of generalized ligamentous laxity (GLL) in cases versus controls

Thirty-three participants (18.33%) in the case group and 16 participants (8.89%) in the control group were found to have GLL. The majority of the participants with GLL in both groups had scores of 4/9 < 5/9 < 6/9. Only four participants (7=3, 8=1, 9=0) among cases and one participant (7=1, 8=0, 9=0) among controls had scores of ≥7/9. Using a cutoff score of ≥5/9, there were 19 participants (10.56%) in the case group and eight participants (4.44%) in the control group (Figure 1).

An odds ratio of 2.30 (using a 2x2 RC table) was calculated between participants with GLL among the cases and controls with a Beighton score of 4/9 with significance p=0.013. A significant odds ratio of 2.5 (p=0.04) was also found between participants with a Beighton score of 5/9. No significant odds ratios were found in a Beighton score of 6/9 and above (Table 2).

**Table 2:** Odds ratio

Beighton score	Cases	Controls	OR ( 95% CI)	p-value
≥4/9	33/180	16/180	2.301 (1.217-4.352)	0.013
≥5/9	19/180	8/180	2.537 (1.081-5.958)	0.044
≥6/9	11/180	4/180	2.864 (0.895-9.170)	0.111
≥7/9	4/180	1/180	4.068 (0.450-36.76)	0.105
≥8/9	1/180	0/180	3.017 (0.122-74.54)	0.501

In an attempt to understand the relationship between GLL and musculoskeletal injuries, mean Beighton scores of the cases and controls were compared for significance; significantly higher mean Beighton score (p=0.018) was found between participants presenting with MSI (cases) and participants without MSI (controls) (Table 3).

**Table 3:** Presence of generalized ligamentous laxity (GLL) in cases vs. controls: arithmetic mean

Description	Cases		Controls		P-value
Beighton score (+/-SD)	1.77 (2.13)		0.92 (1.47)		0.018
Mean Beighton score (+/-SD) males	1.57 (1.93)		0.97 (1.50)		0.021
Mean Beighton score (+/-SD) females	1.88 (2.09)		1.19 (1.72)		0.017
Mean Beighton score (+/-SD)					
15-30 years males	2.20	2.04	1.10	1.69	0.027
15-30 years females	2.67	2.48	1.47	2.06	0.046
31-45 years males	1.67	1.63	0.83	1.26	0.030
31-45 years females	2.10	1.90	1.07	1.55	0.025
46-60 years males	0.83	1.09	0.43	0.73	0.101
46-60 years females	1.17	1.58	0.63	1.00	0.120

**Figure 2:**
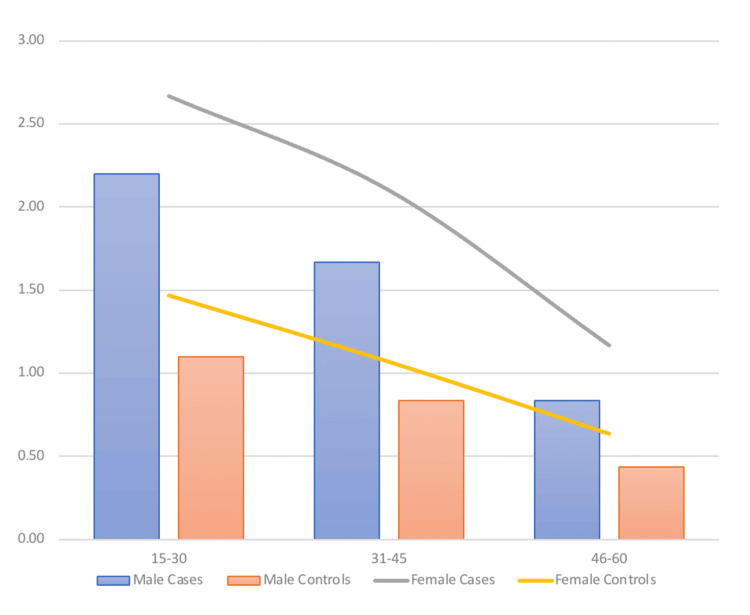
Distribution of the mean Beighton score along age and sex

Similarly, significantly higher mean scores were present in both male (p=0.021) and female cases (p=0.017) compared to controls. Significantly higher Beighton scores were found in ages 15-30 years and 31-46 years in both male and female cases compared to females. No significantly higher scores were found in ages 46-60 years in both sexes (Figure 2).

**Figure 3:**
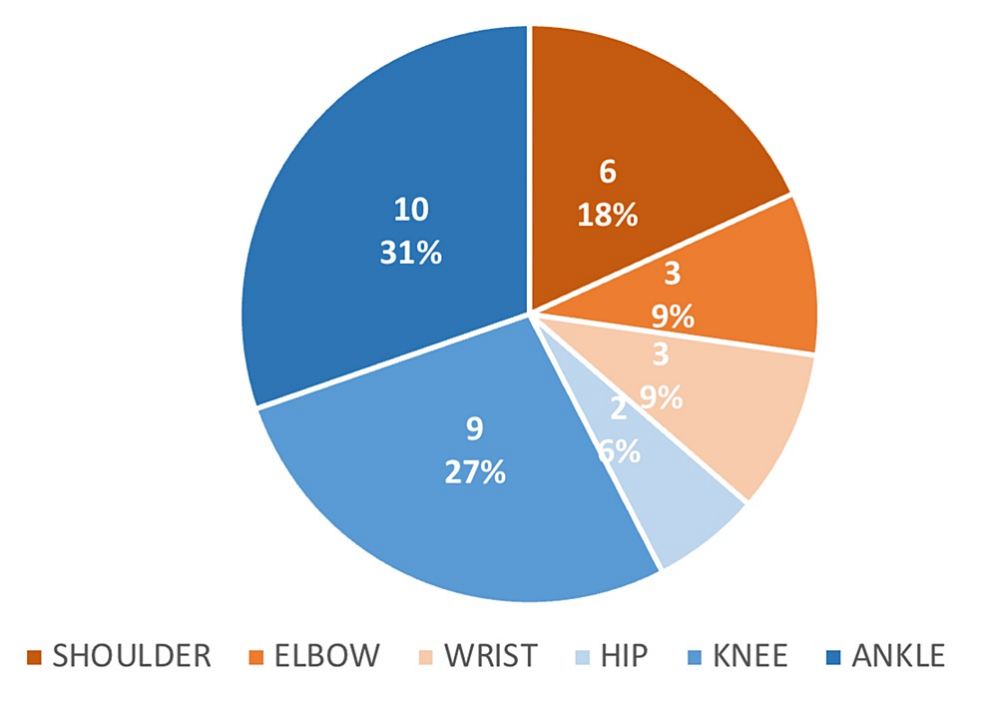
Joints involved in the musculoskeletal injuries in participants with generalized ligamentous laxity (GLL)

There were more lower limb injuries (64%) than upper limb injuries (36%) present among individuals with GLL among participants having musculoskeletal injuries. When joints involved injuries in accordance with sex, females had more involvement of the lower limbs (41%), compared to the upper limbs (25%), which was more than males (25% and 9%, respectively). Figure 3 shows the jointwise distribution of injuries.

## Discussion

GLL is a condition in which most of an individual’s synovial joints move beyond the ‘‘normal’’ limits, with the age, gender, and ethnic background of the individual taken into account. As described earlier, the definition of GLL remains arbitrary, and hence, one of the easiest methods of defining GLL is by utilizing the Beighton score [1,6]. Its advantage is that only a few joints are evaluated, and hence a quick outpatient examination can provide the score without the requirement of any specialized equipment.

A total of 360 participants were examined over the course of this study with a mean age of 37.26 years (SD ±13.8); no significant difference was present between cases and controls in number, sex, mean ages, BMI, and physical activity levels. A total of 49 (13.61%) participants were found to have a Beighton score of 4/9, which was the cut-off defined for the presence of GLL.

Joint hyperlaxity is found to be more common in children. Mobility tends to decrease with an increase in age [11]. A similar finding was also observed in the present study.

A significant odds ratio of 2.3 (p=0.013) was found between the number of cases (33/180) with GLL with a Beighton score of ≥4/9 compared to controls (16/180). It was inferred that 2.3 times more people presenting with musculoskeletal injuries had the presence of GLL than people presenting with other complaints. In view of no significant differences in age, sex, BMI, and activity levels in both cases and controls, it can be extrapolated that persons with GLL have significantly more chances of having musculoskeletal injuries. Furthermore, a significant odds ratio of 2.5 was found between cases and controls with a Beighton score of ≥5/9, which strengthened the association between GLL and MSI.

The odds ratio of this study (2.3) was higher than the combined odds ratio of the meta-analysis by Pacey et al. (1.78), which was probably due to the different scores and cut-off values used in the various studies that were under review [12]. It was comparable to the odds ratio of Nomura et al. (2.4) and lower than that of March, Silman et al. (3.4), and Bin Abd Razak et al. (3.35) [13-15]. Both studies with higher OR were done exclusively in male military recruits who were younger in age, which could have led to an increase in the odds of having an injury in a participant with GLL.

Another method of understanding the relationship between GLL and MSI used in this study was the average Beighton score. The case group presenting with MSI had a significantly higher (p=0.018) mean score of 1.77 (SD±2.13) compared to the control group without MSI, which was 0.92 (SD±1.47). In the present study, a significantly higher mean Beighton score was found in both male and female cases compared to their controls.

Significantly higher mean Beighton scores were also found in the age groups 15-30 years and 31-45 years in both sexes; however, the difference was not significant in the higher age group. There was a decrease in the mean Beighton score with age, and the males of each subgroup also had lower mean scores compared to females.

It was also noticed that the majority of participants in the case group had a higher incidence of lower limb (64%) involvement as compared to upper limb (36%). This was the case across both sexes. A similar finding has been reported by multiple studies; lower limb injuries have been found to be associated more with generalized ligament laxity. The reviews by Pacey et al. and Tingle et al. found that among the studies retrieved, most predominantly explored lower limb injuries and found a significant association for the same [2,12].

Ankle injuries were most commonly reported, followed by knee and shoulder injuries. It may be because of the excessive strain on the knee and ankle as a result of bearing weight and the shoulder being a less congruent joint with stability supplied by a complex ligamentous structure.

When participants were asked about previous injuries, it emerged that approximately half the people with GLL in the control group had a history of MSI in the last two years (but not in the last three months, separate from present complaints about cases). A similar picture emerged from the participants with GLL in the case group, with about half having a history of a previous MSI over the last two years.

Recently, Konopinski et al. studied a cohort study sample of 54 elite-level professional soccer players from an English Premier League club and provided evidence that hypermobile participants had a higher incidence of injuries and were more likely to experience a severe injury, experience a recurrence of injury over the season, and miss more days of training and matches [16]. An association with slower rehabilitation courses for individuals presenting with anterior cruciate ligament (ACL) injuries with GLL was reported by Hardin et al. [17].

As elaborated previously, studies have shown that GLL is associated with many sports injuries. It has also been associated with an increased risk of ACL injuries [18,19], recurrent patellar dislocation [20], and ankle sprains [21]. In the upper extremity, GLL in sportsmen has been associated with multidirectional instability of the shoulder [22] and degeneration of the first carpometacarpal joint [23].

Simmonds and Keer [24] recommended participation in non-contact activities only, while Murray [11] recommended no restrictions for pain-free individuals.

GLL has received less attention in orthopedics, especially in the Indian scenario, and the same is reflected in the dearth of literature in orthopedic journals. However, people with hypermobility syndrome are often first referred to or seen by orthopedic surgeons for acute and chronic musculoskeletal injuries. More research and training are needed to deal with this significant part of the orthopedic patient load.

Patients with GLL usually have a chronic history with no definite history of trauma, and hence, diagnosis may be challenging. A lack of awareness about the relationship between GLL and musculoskeletal injuries may lead to poor outcomes and patient satisfaction [25]. The key to successfully treating a patient with GLL is early identification and initiating prompt treatments. A high degree of clinical suspicion should be maintained, especially in patients presenting with chronic musculoskeletal pain.

Once GLL has been established, targeted exercise regimes should be incorporated. Proprioceptive exercises have been shown to be beneficial in children with GLL and musculoskeletal pain [26].

## Conclusions

This study is the first to assess the relationship between GLL and MSI in an Indian population. The study found that there was a significant prevalence of GLL in the adult population, especially in females compared to males. The younger age group was also comparatively much more involved. It also proved that GLL was more common in patients with MSI and that hypermobile people had a twofold chance of injury compared to the general population. The joints of the lower limb were more frequently involved, probably the weight-bearing joints; the most common were the ankle and knee. People with GLL also had higher chances of repeating injuries.

This study has implications for the prevention of injuries in people with GLL. A screening evaluation of such individuals to identify those with GLL using the Beighton score could be beneficial. For these individuals, specific training techniques aimed at core strengthening and specific muscle and proprioceptive training can be employed to prevent injuries. Though orthopedic surgeons generally primarily manage people with GLL who present with MSI, they do not have a high index of suspicion toward the same. Identifying these individuals and making a diagnosis regarding the same is problematic, but doing so will definitely help these individuals live a pain-free life. Large-scale population studies are suggested for the identification of the true prevalence of GLL.
